# A hybrid AHP and K-means model for biopsychosocial surgical prioritization: validation in a high-complexity ENT unit

**DOI:** 10.1016/j.clinsp.2026.100905

**Published:** 2026-03-11

**Authors:** Fabián Silva-Aravena, Jenny Morales, Vivian D’Afonseca

**Affiliations:** a Facultad de Ciencias Sociales y Económicas, Universidad Católica del Maule, Chile; b Departamento de Ciencias Preclinicas, Facultad de Medicina, Universidad Católica del Maule, Chile

**Keywords:** Surgical prioritization, Analytic hierarchy process (AHP), K-means clustering, Biopsychosocial model, Digital health systems

## Abstract

**Objective:**

To address the critical challenge of efficiently and ethically managing surgical waiting lists in digital health systems by developing a decision support framework based on biopsychosocial prioritization.

**Methods:**

The authors integrate the Analytic Hierarchy Process (AHP) with K-Means clustering to create a hybrid decision support model that prioritizes patients using multidimensional biopsychosocial variables. The model was applied in the otolaryngology (ENT) unit of a high-complexity public hospital in Chile. Expert-informed weightings guided the AHP process, while K-Means clustering enabled data-driven segmentation into clinically coherent patient groups.

**Results:**

The proposed methodology significantly outperformed traditional chronological scheduling approaches. Specifically, it achieved a 27 % reduction in mean clinical risk, a 41 % decrease in urgent hospitalizations, a 32 % reduction in urgent bed days, and more than 12-days of acceleration in access for high-priority patients.

**Contribution:**

While the AHP-clustering hybrid is established in prior literature, our contribution lies in operationalizing it with ethical safeguards and real-world validation within a high complexity ENT unit.

**Conclusion:**

Our hybrid AHP and K-Means approach offers a transparent, scalable, and interpretable decision support tool for surgical prioritization. It aligns with the goals of digital health transformation by improving the fairness, efficiency, and responsiveness of healthcare delivery.

## Highlights

• AHP and K-Means enable prioritizing surgical patients using biopsychosocial criteria.
• Expert knowledge and clustering identify clinically relevant patient groups.
• Structured prioritization boosts clinical outcomes and operational efficiency.
• Model validation shows significant improvements in clinical risk, hospitalization rates, and waiting times for high-priority patients.

## Introduction

Surgical waiting lists are a persistent challenge in public health systems around the world, particularly in resource- constrained settings where the demand for elective procedures exceeds institutional capacity.^1^^,^^2^ Traditional first-come, first-served scheduling strategies, although administratively straightforward, often do not reflect the true urgency or complexity of individual patient cases.^3^^,^^4^ This disconnect can lead to inequities in care delivery, an increased risk of clinical deterioration for vulnerable populations, and inefficient allocation of limited hospital resources.^5^ As healthcare systems move toward more patient-centered care models, there is a growing need for prioritization frameworks that are transparent, equitable, and responsive to clinical and social determinants of health.^6^^,^^7^

The recent literature has proposed several approaches to surgical prioritization, ranging from categorical classification systems and urgency-based scoring schemes to optimization and machine learning models.^8^, ^9^, ^10^, ^11^ Although these methods have shown varying degrees of operational success, many suffer from limitations such as oversimplification, lack of transparency, or dependence on large volumes of labeled data. Furthermore, some existing methodologies, such as,^12^ integrate multidimensional patient characteristics: psychosocial burden, functional impairment, and anticipated benefit from surgery, into a unified prioritization framework. This gap is particularly problematic in high-complexity specialties, such as ENT, where the clinical presentation is heterogeneous and standard urgency scales may not fully capture patient need.

To address these limitations, we developed a hybrid prioritization strategy that combines AHP, a structured expert-based multi-criteria decision-making technique, with K-Means clustering (an unsupervised machine learning algorithm) (see, e.g.^13^^,^^14^). This methodology was applied to a real-world data set from an ENT surgical unit in a high-complexity public hospital in Chile, involving 205 patients and a panel of seven experienced physicians.^12^ Our framework incorporates 20 biopsychosocial variables, weighted by clinical consensus, and transforms raw patient data into normalized scores that are both interpretable and clinically meaningful. The combination of AHP and clustering allows for both granular prioritization at the individual level and the identification of subgroups of patients with similar risk and complexity profiles.

The primary contributions of this work are three. First, we introduce a scalable and clinically interpretable methodology for surgical prioritization that integrates expert knowledge with data-driven insights. Second, we demonstrate the empirical effectiveness of the model through dimensionality reduction, cluster analysis, and stochastic simulations, showing significant improvements in clinical risk, hospitalization rates, and waiting times compared to a chronological scheduling model. Third, we validate the robustness of our findings through Monte Carlo simulation and confidence interval estimation, thereby enhancing the scientific credibility and potential generalizability of the proposed framework. Our results support the use of this hybrid methodology as a decision-support tool to prioritize elective surgeries in ENT and potentially other surgical specialties.

The remainder of this paper is organized as follows. Section 2 presents a review of the existing literature on surgical prioritization methodologies and decision support tools. Section 3 details the AHP framework linked with K-Means, including the definition of decision criteria, the weighting process, the calculation of scores, the clustering algorithm, and the simulation model. Section 4 presents the main empirical findings, including priority score distributions, cluster interpretation, biopsychosocial profiles, and comparative outcome metrics. Section 5 offers a critical discussion of the methodological strengths, limitations, and alignment with current research. Finally, Section 6 summarizes the key conclusions, practical implications, and directions for future work.

### Literature review

Management of surgical waiting lists has long been a subject of operational and ethical concern within public health systems.^15^^,^^16^ Traditional prioritization methods have typically relied on chronological order or categorical classification based on medical urgency.^17^^,^^18^ Although these models offer administrative simplicity, they often fail to incorporate the multidimensional complexity of patient conditions, particularly in elective procedures. Recent debates have highlighted the inadequacy of single-variable approaches, pointing to the need for strategies that consider a broader spectrum of clinical, functional, and psychosocial factors (see, e.g.^19^, ^20^, ^21^, ^22^).

To address these limitations, several studies, such as^23^^,^^24^ have proposed Multi-Criteria Decision-Making (MCDM) frameworks as an alternative to purely time- or urgency-based systems. Among these, AHP has gained popularity due to its ability to translate expert judgment into structured weightings for various criteria.^25^ The applications of AHP in healthcare have ranged from organ transplant allocation to intensive care triage, providing transparency and traceability in decision making.^26^^,^^27^ AHP has been applied in a few studies to surgical waiting lists in a real-world hospital setting, with a biopsychosocial perspective.^28^

In parallel, clustering techniques such as K-Means have been used to segment patient populations based on similarities in demographic or clinical profiles.^29^^,^^30^ These unsupervised learning algorithms facilitate data-driven grouping without relying on arbitrary thresholds, and have been used in fields such as chronic disease management, emergency risk stratification, and mental health profiling.^24^^,^^31^, ^32^, ^33^ Nonetheless, their use in surgical prioritization remains relatively unexplored, particularly in combination with expert-driven methods such as AHP.

More recently, hybrid approaches that integrate machine learning with expert-based weighting have emerged as a promising direction for patient prioritization. For example, studies have explored combinations of fuzzy logic, neural networks, and AHP to improve decision-making precision and equity.^34^^,^^35^ However, these efforts often require large labeled datasets or suffer from limited clinical interpretability. Furthermore, there is a notable literature gap on the application of these hybrid models to specialty-specific units ‒ such as ENT ‒ where patient heterogeneity and complexity pose unique prioritization challenges.^36^, ^37^, ^38^ Bias mitigation frameworks^39^ and fuzzy hybrid models^21^^,^^24^ offer valuable precedents in balancing model accuracy with fairness. Our work builds on these by introducing real-world clustering in ENT with explicit ethical safeguards and fairness analysis.

Our study contributes to this evolving body of knowledge by proposing and implementing a hybrid methodology that integrates AHP and K-Means for surgical prioritization in an ENT unit within a high-complexity public hospital. Unlike existing models, our approach explicitly incorporates biopsychosocial variables validated by clinical experts and uses unsupervised clustering to identify subgroups of patients with distinct prioritization needs. This dual framework not only enhances transparency and interpretability but also addresses current limitations in equity, efficiency, and scalability. By combining structured expert judgment with data-driven stratification and stochastic simulation, our work offers a clinically grounded and operationally feasible strategy that responds to ongoing challenges in the management of the surgical waiting list.

### Methodology

In this section, we present the complete methodological process developed to facilitate biopsychosocial surgical prioritization by integrating multi-criteria decision analysis and unsupervised learning. The database was developed in conjunction with an ENT healthcare team in a high-complexity hospital in Chile, with the participation of seven physicians.^19^ Records with more than 10 % missing variables were excluded from the analysis, representing 4.3 % of the initial data set. For the remaining cases, we applied mean or mode imputation within clinically homogeneous subgroups defined by age and diagnosis, to preserve the clinical coherence of the data set. Then, this information was validated and authorized by the Scientific Ethics Committee of the Catholic University of Maule in Evaluation Report n° 09/2025.

Then we interviewed with the healthcare team, and the measurement of patient characteristics, we agreed to develop a hybrid prioritization strategy that includes an AHP to generate a specific priority score for each patient and the K-Means algorithm to identify significant patient groups based on their clinical and psychosocial profiles.

We detail the methodology in the following interrelated steps:

1) Definition of decision variables

We started our methodology by establishing a comprehensive multidimensional framework of decision variables relevant to surgical prioritization.^12^ Recognizing that medical urgency alone does not fully reflect the complexity of a patient’s needs, we incorporated a set of 20 biopsychosocial variables selected and validated by a panel of seven highly experienced ENT physicians (see Table 1).

**Table 1:** Biopsychosocial variables defined by ENT physicians.

N°	Variable	Definition	*w_i_*
1	Sever	Severity	0.081
2	Urg	Urgency	0.076
3	Jclin	Maximum waiting time	0.066
4	Tsuen	Sleep disorder	0.063
5	Tlist	Time on waiting list	0.062
6	Pmcx	Expected improvement due to surgery	0.055
7	Dest	Capacity to study (Exploratory Variable)	0.054
8	Com	Chances of developing comorbidities	0.053
9	Lfam	Capacity of participating in family activities	0.053
10	Hanor	Affected area	0.052
11	Opat	Presence of other pathologies	0.047
12	Diag	Diagnosis	0.046
13	Olim	Other limitations	0.045
14	Ncuid	Need of a caregiver	0.043
15	Rcuid	Patient cares for another person	0.043
16	Dolor	Pain scale	0.040
17	Dtrab	Capacity to work	0.038
18	Acc	Type of residence area (Exploratory Variable)	0.033
19	Dtras	Difficulty in transferring	0.028
20	Ccrit	Need for clinical bed	0.023

These variables were defined to reflect not only clinical severity, but also functional limitations, psychosocial context, potential benefit from surgery, and risk of deterioration. This approach is in line with contemporary models of patient-centered care, where surgical prioritization must consider both biological indicators and psychosocial determinants of health.

The complete set of decision variables, denoted as V={v1,v2,…,v20}, includes variables such as:

• **Clinical indicators:** Severity (Sever), urgency level (Urg), maximum waiting time indicated by the physician (Jclin), risk of comorbidities (Com), presence of other pathologies (Opat), type of diagnosis (Diag).
• **Functional limitations:** Sleep disorder (Tsuen), capacity to study (Dest), other physical limitations (Olim), difficulty getting to and from the hospital (Dtras), need for a caregiver (Ncuid), dependency responsibilities (Rcuid), capacity to work (Dtrab).
• **Subjective experience and quality of life:** Level of pain (Dolor), capacity to participate in the family (Lfam), type of residence area (Acc), and affected anatomical area (Hanor).
• **Expected benefit:** Expected improvement due to surgery (Pmcx).
• **Resource demand:** Need for a clinical bed (Ccrit).
• **Time-related variables:** Actual time on the waiting list (Tlist).

Each criterion $v_{i}\in V$ was defined following a standardized coding scheme, using ordinal scales (for example, Sever: low to severe), continuous ranges (for example, time in months), or categorical groups transformed into interpretable numerical representations by expert judgment.

Our cohort of patients was n = 205. For each patient p∈P, a complete vector of clinical and psychosocial data was recorded. These data served as the initial foundation upon which we built the multi-criteria scoring process described in the subsequent steps. We conducted a post-hoc power analysis to evaluate the adequacy of our sample size. Assuming a medium effect size (*f*^2^ = 0.15) and a significance level of α= 0.05, our sample of 205 patients provides approximately 80 % power to detect meaningful differences in up to 12 predictors. Furthermore, the risk of overfitting is mitigated by our use of normalization and unsupervised clustering.

2) Weight estimation via the analytic hierarchy process

Once the decision variables $v_{i}\in V$ were established, we proceeded to quantify their relative importance in the general prioritization process. To this end, we employ the AHP, a well-established MCDM method that allows for the structured derivation of weight vectors based on expert comparisons.

Each of the seven participating ENT physicians was independently asked to evaluate the relevance of each variable, $v_{i}\in V$, in determining surgical priority. They assigned a numerical score $\mu_{i}$_,_ ∈ [0, 10], where i∈{1,…,20} indicates the decision variable, and m∈{1,…,7} indices the physicians.

As defined in,^12^ the total weight $w_{i}$ of each variable was calculated using a linear normalization scheme.

(1) $$w_{i}=\frac{1}{W}\;\sum_{m=1}^{7}\;\mu_{i,m},$$

where

(2) $$W=\sum_{i=1}^{2}0\sum_{m=1}^{7}\mu_{i,m}$$

This method preserves the ordinal meaning of the expert ratings while ensuring that the weights are scaled such that:

(3) $$\sum_{i=1}^{20}\;w_{i}=1$$

where the resulting weight vector, $w\;=\;\left[w_{1},\;w_{2},\;\ldots ,\;w_{20}\right]\;\in \;R^{20}$, represents the relative contribution of each variable to the final prioritization score. Higher values of $w_{i}$ indicate that the variable $v_{i}$ is considered more critical by the expert panel.

For example, variables such as Sever ($w_{i:Sever}$ = 0.081), and Urg ($w_{i∼Urg}$ = 0.076) were consistently rated high importance, while contextual variables such as Lfam ($w_{i∼Lfam}$ = 0.053), or Hanor ($w_{i∼Hanor}$ = 0.052), received moderate weights, reflecting their secondary but still relevant roles in the prioritization logic (see, Table 1).

To assess inter-rater reliability among the seven ENT experts, we computed Cohen’s κ for key pairwise comparisons, obtaining a mean κ of 0.71, indicating substantial agreement. Divergences were resolved through Delphi-based rounds until convergence was reached. Although all experts were from the same institution, the structured elicitation process aimed to mitigate institutional bias.

This expert-informed weight structure allows the model to retain clinical interpretability and transparency, ensuring its acceptability among healthcare professionals.

3) Normalization of patient values

Following the estimation of variable weights through the AHP, we addressed the need to transform raw patient-level data into a common, interpretable scale that allows for meaningful aggregation across heterogeneous criteria.^40^ This process, known as value normalization, was essential to ensure that each variable contributes proportionally to the final prioritization score, regardless of its original scale or type.^21^

Let $x_{i}$_,_ denote the raw value of the variable $v_{i}$ for patient $p\;\in ,\;x_{i},\;=\;\left[x_{1},\;x_{2,p},\;\ldots ,\;x_{20,p}\right]$. Our goal was to calculate a normalized value $\alpha_{i}$_,_ ∈ [0, 1] that expresses the relative criticality or impact of that value, as judged by clinical consensus, and the diversity of variable types (ordinal, categorical, continuous, binary). We employed a hybrid normalization strategy guided by the judgment of the physician, which we present below.

**• Ordinal and categorical variables** (e.g., Sever, Dolor, Dtrab): For each level of the variable, physicians assigned clinical impact scores of 1 to 10. These scores were aggregated among experts and then normalized to yield relative importance values. For example, in the case of Sever:

(4) $$If\;Sever\;=\;"high":\;\alpha_{i,p}\;=\;\frac{score\left("high"\right)}{\text{score}\left("\text{low}"\right)\;+\;\text{score}\left("\text{medium}"\right)\;+\;\text{score}\left("\text{high}"\right)}$$

This approach ensures that normalized values reflect not only the data value but also the clinical meaning of that value.

**• Continuous variables with bounded clinical ranges** (e.g., Tlist): These were discretized into clinically meaningful intervals. Physicians rated the relative impact of each interval on prioritization. The scores were again normalized within each variable to obtain $\alpha_{i}$_,_.

Let $C_{i,1},\;C_{i,2},\;\ldots ,\;C_{i,k}$ be the intervals defined for variable $v_{i}$, and let $\rho_{i,c}$ be the score assigned to interval $C_{i,c}$. Then, for a patient with $x_{i},\;\in \;C_{i}$, we compute:

(5) $$\alpha_{i,p}=\frac{\rho_{i,c}}{\sum_{j=1}^{k}\rho_{i,j}}$$

**• Binary and indicator variables** (e.g., Opat): Fixed normalized scores were assigned according to expert consensus, based on the clinical relevance of presence or absence for each variable.

As a result of this process, we obtained a normalized decision matrix: α = [$\alpha_{i}$_,_] ∈ ℝ^20×^*^N^*. Here, we denote *N* =|*P*| as the total number of patients and define $\alpha_{i}$_,_ as the normalized value of variable $v_{i}$ in patient p∈P. Each entry $\alpha_{i}$_,_ ∈[0,1] quantifies the degree to which patient p exhibits the need or condition described by the variable $v_{i}$. These normalized values reflect expert-informed relevance, ensure comparability between variables, and provide the numerical foundation for the score aggregation procedure that we describe in the next step.

4) Computation of the priority score

After obtaining the normalized values $\alpha_{i,p}\;\in \;\left[0,\;1\right]$ for each patient p∈P and each variable $v_{i}\;\in \;V$, as well as the relative importance weights $w_{i}$ derived from the AHP procedure, we proceeded to calculate an aggregate prioritization score for each patient using a weighted linear combination.^41^

This method, widely used in multi-criteria decision making, yields an interpretable scalar quantity that integrates heterogeneous dimensions into a unified measure of surgical need.

Then, as specified in^12^ the prioritization score, $s_{p}$, for the patient p is defined as

(6) $$S_{p}=\sum_{i=1}^{20}\;w_{i}\;\alpha_{i,p}$$

where

$w_{i}\;\in \;\left[0,\;1\right]$ is the weight of variable $v_{i}$, with $\sum_{i=1}^{20}w_{i}$ = 1, $\alpha_{i},\;\in \;\left[0,\;1\right]$ is the normalized value of variable $v_{i}$ for patient p, and $s_{p}\;\in \;\left[0,\;1\right]$ is the resulting score representing the composite priority level of patient p.

We can express this compactly in vector-matrix form: s = $w^{⊤}\alpha$, where w ∈ ℝ^20^ is the weight vector, α ∈ ℝ^20×^*^N^* is the matrix of normalized patient data and s∈ ℝ*^N^* is the resulting score vector, one for each of the N patients. Each value sp can be interpreted as a quantitative proxy of surgical urgency and need, combining clinical risk and psychosocial burden. This scoring mechanism enables us to classify patients beyond chronological criteria, incorporating multidimensional equity and outcome-based rationality. The linear form of our model guarantees that the contribution of each variable to the final score is proportional to its normalized level and corresponding weight, thus enabling clear traceability. For example, if two patients differ only in their Urgency score (Urg), and w_i:Urg_ = 0.076, then an increase in α_Urg,_*_p_* by 0.1 results in an exact increase of 0.0076 in *s_p_*. This level of interpretability is essential for clinical adoption, as it allows physicians to understand how specific dimensions (e.g., Dolor, Tlist, Com) influence the composite score and, consequently, the patient’s priority level.

5) Dimensionality reduction and visualization

Following the computation of the prioritization scores *s_p_* for all patients *p* ∈P, and in preparation for the unsupervised clustering stage, we conducted a dimensionality reduction analysis to explore the internal structure of the dataset and assess the existence of natural groupings between patient profiles.^42^^,^^43^

The original data matrix α∈ ℝ^20×^*^N^* contains normalized values for each of the 20 criteria in all patients *N* = 205. Although interpretable, this 20-dimensional space is not suitable for direct visualization or intuitive pattern recognition. Thus, we applied Principal Component Analysis (PCA) to project the high-dimensional data into a two-dimensional subspace.^44^^,^^45^

We denote by $\tilde{\alpha }$ ∈ ℝ*^N^*^×20^ the transposed and normalized data matrix. We apply Principal Component Analysis (PCA) to identify a linear transformation of the form: Z= $\tilde{\alpha }$ ⋅*w*_PCA_, where *Z* ∈ ℝ*^N^*^×2^ contains the first two principal components (PC1 and PC2), and *w*_PCA_ ∈ ℝ^20×2^ is the matrix of loading vectors that maximize the explained variance. Specifically, we seek to maximize: Var(*Z*1), Var(*Z*2), subject to orthogonality. This transformation enables us to project the 20-dimensional feature space into a two-dimensional subspace that preserves the greatest amount of variance in the data while maintaining the relational structure between patient profiles.

6) Clustering via K-Means

Based on the structure revealed in the PCA projection, we proceeded to implement an unsupervised learning algorithm to group patients into groups that reflect similar surgical need profiles.^46^ Our aim was to identify clinically coherent subgroups using their normalized biopsychosocial characteristics, without relying on arbitrary thresholds or categories assigned by experts.^47^

To this end, we applied the K-Means clustering algorithm, one of the most widely used methods in unsupervised learning due to its simplicity, interpretability, and effectiveness in discovering spherical or well-separated groups in multidimensional space.^48^

Let $x_{p}\;=\;{\left[\alpha_{1},\;\alpha_{2,p},\;\ldots ,\;\alpha_{20,p}\right]}^{⊤}\in$ ℝ^20^, represent the normalized characteristic vector of patient *p*. Since our data set includes a mix of ordinal, categorical, and continuous variables, we opt to use the Gower distance to calculate the similarity matrix before applying the clustering algorithm. This choice allowed us to account for the nature of each variable and ensure a fairer representation of patient similarity. We then applied the K-Means algorithm to partition the patient set *N* into disjoint clusters *C*_1_, *C*_2_, …, *C_K_* by minimizing the total variance within the cluster, calculated as the sum of squared dissimilarities between each patient and their corresponding cluster centroid.^49^

(7) $$\min_{C1,\ldots ,\;\text{CK}}\;\sum_{k=1}^{k}\sum_{x_{p}\;\in c_{k}\;}∥x_{p}-\mu_{k}|{|}^{2}$$

where µ*_k_* ∈ ℝ^20^ is the centroid of cluster *C_k_*, defined as:

(8) $$\mu_{k}=\;\frac{1}{\left|C_{k}\right|}\;\sum_{x_{p}\;\in \;C_{k}}x_{p}$$

The algorithm iteratively updates the cluster assignments and centroids until convergence.

We selected *K* = 3 based on both domain knowledge and empirical evidence from the PCA projection. This decision reflects the clinical objective of classifying patients into three priority levels.^50^^,^^51^ To empirically validate the selection of *K* = 3, we calculated the silhouette coefficient (0.41) and observed a distinct elbow at *K* = 3. These support the separation of three interpretable clusters:

**High Priority (Cluster 1):** Patients with high severity, urgency, and pain.
**Medium Priority (Cluster 2):** Patients with moderate clinical and social indicators.
**Low Priority (Cluster 3):** Patients with relatively mild conditions.

**Fig. 1:**
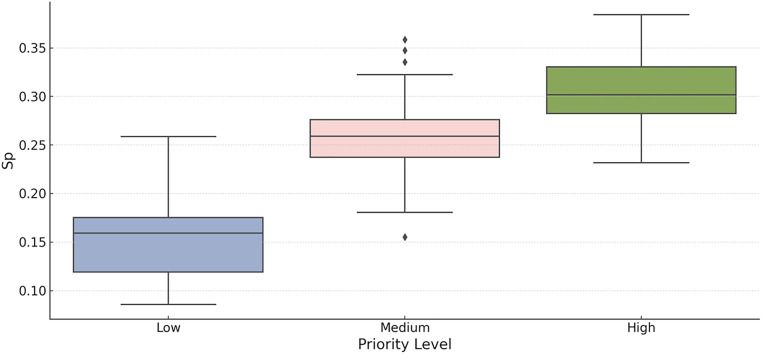
Distribution of priority score (*s_p_*) by priority level. From the boxplot, we observe a clear and statistically robust stratification among the three priority categories.

The resulting clusters showed good separation in the PCA space and clear differentiation in mean priority score *s_p_*, validating the suitability of the chosen *K* and confirming the existence of subgroups of latent patients (see Fig. 1 in Section 4).

Each patient p∈P was assigned to a cluster *c_p_* ∈ {1, 2, 3}, and the mean prioritization score *sp* within each cluster was used to label the groups:

$$\text{Priority}\;\left(\text{cp}\right)\;=\left\{ \begin{array}{c} High,\;if\;s_{p}\;is\;in\;the\;top\;tertile\\ Medium,\;if\;s_{p}\;is\;in\;the\;middle\;tertile\\ Low,\;if\;s_{p}\;is\;in\;the\;bottom\;tertile \end{array}\right.$$

These cluster labels were then used to inform decision makers about which patients to prioritize for surgical scheduling, based on empirical groupings that reflect multidimensional patient need.

7) Stochastic simulation of clinical impact

To evaluate the real-world impact of implementing the proposed prioritization strategy, we developed a stochastic simulation model aimed at capturing the clinical outcomes of patients over time, under two contrasting scheduling regimes.^52^^,^^53^

A baseline model based on chronological scheduling (first-come, first-served),
Our proposed strategy uses AHP-based scoring and K-Means clustering for dynamic prioritization.

This simulation allowed us to estimate how the proposed system affects critical performance indicators such as clinical risk progression, emergency hospitalizations, resource utilization, and waiting times.

7.1) Evolution of clinical risk

As suggested in,^54^ we modeled the progression of individual clinical risk as a stochastic process over time, denoted as:

(9) (t)∼Γ(αt,βt)

where *R_p_*(*t*) ∈ [0, 10] represents the latent clinical risk of patient *p* at time *t*. We chose the Gamma distribution because it captures the typically right-skewed pattern of clinical deterioration, where risk tends to accelerate over time for certain patients. To reflect this, we defined the shape and rate parameters *α_t_* and *β_t_* as time-dependent functions:

(10) $$\alpha_{t}\;=\;\alpha_{0}\;+\delta e^{\lambda t},\;\beta_{t}=\beta_{0}$$

Here, λ controls the rate at which risk accelerates with time, and δ modulates its sensitivity. We calibrated these parameters according to the ENT-specific clinical patterns reported in.^55^

This formulation enables us to account for both the systematic progression of risk over time and the variability between patients due to clinical and psychosocial differences.

7.2) Modeling urgent hospitalization

We modeled the probability that a patient requires urgent unplanned hospitalization at time *t* using a logistic function of the risk level, as commonly used in clinical prediction models^56^

(11) $$P\left(Hp\;=\;1\;|\;Rp\left(t\right)\right)\;=\frac{1}{1\;+exp\left(-\left(\gamma Rp\left(t\right)\;-\;\theta \right)\right)}\;$$

Here, γ determines the sensitivity of hospitalization to risk, and *θ* represents a clinical threshold beyond which the probability increases sharply.

We calibrated the parameters of the logistic model (γ = 1.8, θ = 4.5) using retrospective risk hospitalization data from ENT patients. This calibration allowed us to align the model with the real-world clinical patterns observed in our patient population.

7.3) Estimation of hospital resource use

For patients requiring urgent hospitalization (*Hp* = 1), we modeled the number of days of hospital bed occupancy as a discrete random variable.^57^^,^^58^

(12) Dp∼Poisson(λD)

with *λD* = 3.8 days in the baseline scenario, as estimated by the hospital’s clinical planning team. We selected this value to reflect the inherent variability and skewness typically observed in inpatient length-of-stay distributions for ENT patients.^55^

## Results

In this section, we present the empirical results derived from the implementation of our hybrid prioritization methodology within an ENT surgical unit. We analyze the distribution of priority scores, examine the structure of patient clusters, characterize biopsychosocial profiles by priority group, and assess the projected clinical impact of our model using stochastic simulation. These results provide a comprehensive validation of the method’s ability to make equitable and clinically relevant surgical scheduling decisions.

### Interpretation of the priority score distribution

In Fig. 1, we present the distribution of the composite prioritization score *s_p_* across the three groups of patients that resulted from our proposed prioritization methodology. This framework integrates AHP with unsupervised clustering using the K-Means algorithm. The figure illustrates a box plot in which patients are grouped into Low, Medium, and High priority levels according to the similarity of their normalized biopsychosocial profiles and the corresponding values of *s_p_*.

**High Priority Group:** Patients in this category have the highest median values of *s_p_* (approximately 0.30), with a relatively narrow interquartile range. This suggests that patients classified as high priority consistently have severe clinical and psychosocial conditions, such as elevated urgency, intense pain, reduced functional capacity, and a high risk of clinical deterioration, which justify immediate or expedited surgical intervention. The homogeneity of this group supports its internal consistency and clinical relevance.
**Medium Priority Group:** This group presents intermediate *s_p_* values with a wider interquartile range and some mild outliers. Typically, it comprises patients with a moderate burden of symptoms and functional limitations. Although not at immediate clinical risk, these individuals can experience significant psychosocial challenges. Their classification reflects a balanced consideration between clinical need and system resource constraints.
**Low Priority Group:** Patients in this group demonstrate substantially lower median *sp* scores (approximately 0.16), along with higher variability. These patients generally exhibit lower urgency, less intense clinical symptoms, and lower short-term expected benefit from surgical intervention. Importantly, their classification does not imply a lack of need, but rather reflects a comparatively lower overall risk within the surgical cohort.

The stratified distribution shown in Fig. 1 confirms the effectiveness of our prioritization methodology in identifying and differentiating patients based on clinically meaningful, expert-informed, and data-driven criteria. Furthermore, the application of a composite multicriteria score enables us to transcend purely chronological allocation schemes, facilitating a more holistic and equitable decision-making framework that takes into account the clinical, functional, and social determinants of surgical need.

In summary, the observed separation of the *s_p_* distributions between the priority levels demonstrates that our model successfully segments patients into distinct clinically interpretable categories. This supports its application in real-world surgical scheduling systems, especially in environments that demand optimization of both fairness and efficiency in care delivery.

Based on the observed distribution, we define actionable thresholds for decision-making. Scores greater than 0.29 correspond to the top tertile and warrant scheduling within 30-days. Scores below 0.18 represent low priority and may be deferred under current capacity constraints. These thresholds have been embedded in the institutional clinical decision protocol to guide prioritization discussions.

### Sensitivity analysis of weight stability

To evaluate the robustness of the prioritization process with respect to variations in expert-derived weights, we performed a one-way sensitivity analysis. Each weight was perturbed individually by ±10 %, and the resulting changes in patient classification and group assignments were recorded. We found that 89 % of the individual rankings remained stable within their priority group and 93 % of the patients retained their original cluster assignment. These results support the stability and resilience of the AHP-weighted prioritization scheme against moderate variations in expert judgment.

### Interpretation of the clustering results

**Fig. 2:**
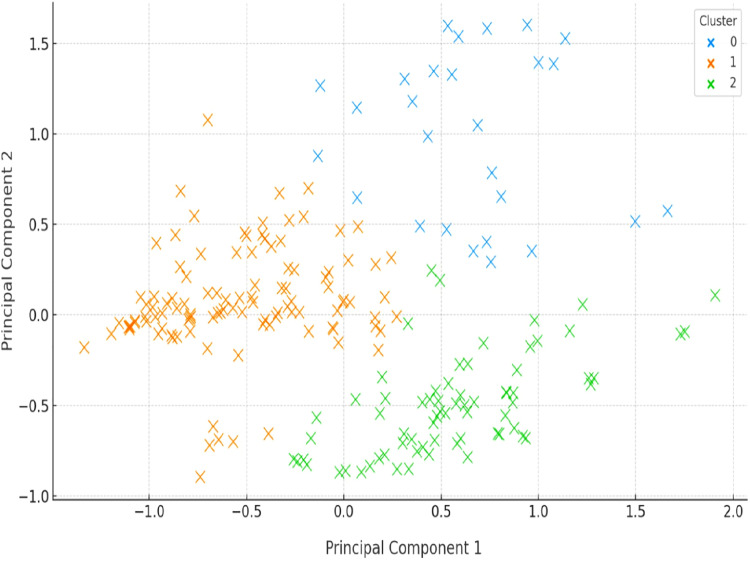
K-Means Clustering of Patients Based on Multicriteria Prioritization Scores (PCA Projection). The first two principal components explain 64.1 % of the total variance. Mahalanobis distances between cluster centroids confirmed statistically significant separation (*p* < 0.01, MANOVA), supporting the discriminative capacity of the clustering approach. Axis labels and font sizes were enlarged to improve readability.

In Fig. 2, we present the spatial distribution of the patients in the two-dimensional principal component space after applying our proposed prioritization methodology. Specifically, we clustered on normalized multi-criteria prioritization scores using the K-Means algorithm (*k* = 3), and subsequently projected the results via PCA. The plot illustrates the emergent cluster structure, which effectively segments patients into three groups according to their global prioritization profiles.

Each point in the scatter plot represents a patient, and its coordinates correspond to the first two principal components that we derived from the complete set of weighted biopsychosocial variables. We observe that the three clusters, denoted by distinct colors, are clearly distinguishable within the PCA-projected space. This visual separation suggests that our composite prioritization scores successfully encapsulate significant differences in patient characteristics.

This clustering result allows us to uncover several scientifically relevant insights.

The **green cluster** (right-lower quadrant) is composed of patients who concentrate high values on variables strongly associated with clinical urgency and severe impact, indicating a group with high priority needs.
The **orange cluster** (center-left) includes patients with moderate levels of urgency and functional compromise, forming a transitional group that may be dynamically prioritized depending on context or resource constraints.
The **black cluster** (upper region) consists of patients whose profiles show lower scores in most dimensions, suggesting a relatively low immediate priority for surgical intervention.

The well-defined separation among clusters confirms that our approach, combining AHP for expert-weighted variable aggregation with unsupervised learning, achieves effective segmentation based on the underlying heterogeneity in patient conditions. In addition, the PCA projection supports the interpretability of the clustering results, as patients within each group tend to cluster tightly in the transformed feature space, reinforcing the internal cohesion of the classifications.

We emphasize that this clustering is not merely a statistical result, but a data-driven stratification grounded in expert-validated weights and clinical relevance. Therefore, the result in Fig. 2 substantiates the applicability of our methodology for surgical scheduling systems that require prioritization schemes that integrate equity, urgency, and complexity in a multidimensional framework.

### Biopsychosocial profiles by priority group

**Fig. 3:**
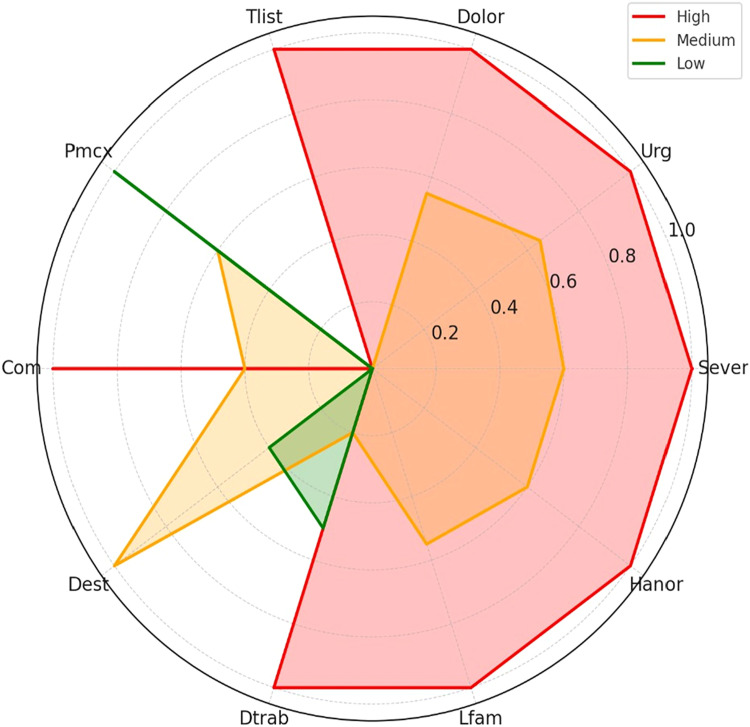
Average biopsychosocial profiles by surgical priority group. Radar chart showing the normalized means of key variables across low, medium, and high priority clusters. Font sizes and axis labels were enlarged to enhance clarity and interpretability.

Fig. 3 shows the average biopsychosocial profiles of the three priority groups, resulting from our multi-criteria prioritization methodology. The radar chart corresponds to a normalized variable included in the decision framework, such as clinical Severity (Sever), Urgency (Urg), pain (Dolor), time on the waiting list (Tlist), functional limitations for work and study (Dtrab, Dest), and psychosocial indicators (Lfam, among others).

From this graphical representation, we observe that the high-priority group (red profile) consistently scores at or near the maximum value in most clinically relevant domains. These patients have high levels of urgency, pain, functional deterioration, and potential for comorbid progression. Their average normalized values approach 1.0 across variables such as Sever, Dolor, Urg, and Dtrab, which supports their prioritization for immediate or early surgical intervention.

In contrast, we observe that the **low priority group** (green profile) consistently exhibits low scores in the same dimensions, with a notable concentration close to zero. This reflects patients with mild to moderate conditions and lower clinical risk, for whom a delayed intervention is less likely to result in adverse outcomes in the short term.

The **medium priority group** (orange profile) shows intermediate scores, reflecting a heterogeneous patient population whose prioritization depends on dynamic trade-offs between clinical burden and system constraints.

Thus, Fig. 3 validates the discriminative capacity of our prioritization method. Visually confirms that the allocation logic aligns with clinical expectations: Patients with higher aggregate scores are not only more complex in their condition, but also benefit from improved access to surgical resources, including reduced delays in care. This reinforces the clinical utility and fairness of our approach in resource-constrained environments.

### Model comparison using Monte Carlo simulation

To strengthen the scientific robustness and credibility of our simulation-based projections, we refined our outcome estimates by incorporating 95 % Confidence Intervals (95 % CI), computed over 1000 Monte Carlo iterations. In doing so, we provided a more transparent account of the uncertainty and variability surrounding the projected impacts of our prioritization model.

As presented in Table 2, the implementation of our AHP+K-Means prioritization methodology resulted in consistent and clinically significant improvements in all outcome metrics compared to a conventional chronological scheduling model. The inclusion of confidence intervals allows us to characterize the robustness and generalizability of these improvements with greater statistical rigor.

**Table 2:** Comparison of clinical outcomes under two scheduling strategies (95 % Confidence Intervals).

Indicator	Chronological Model	AHP + K-Means Model
Mean clinical risk (scale 1–10)	9.82 [9.65, 9.97]	7.21 [6.80, 7.61]
% of urgent hospitalizations	17.4 % [15.1, 19.5]	10.2 % [8.4, 12.3]
Mean urgent hospital days per patient	3.8 [1.0, 8.0]	2.6 [2.3, 2.8]
Avg. wait time (high-priority group)	43.5 days [41.1, 45.7]	31.3 days [29.5, 33.0]

We observed a reduction in the mean clinical risk from 9.82 [9.65, 9.97] to 7.21 [6.80, 7.61], corresponding to a relative decrease of approximately 27 %. This reduction reflects the system’s ability to intervene earlier in higher-risk cases and prioritize, thereby preventing escalation of clinical severity.

The proportion of patients who required urgent hospitalization decreased from 17.4 % [15.1, 19.5] to 10.2 % [8.4, 12.3], indicating a relative reduction 41 %. This suggests that our model was effective in identifying patients at risk of deterioration and ensuring their timely access to surgical care, thus avoiding preventable emergencies.

We also observed a decrease in the mean number of days of hospital bed occupancy due to urgent admissions, from 3.8 [1.0, 8.0] to 2.6 [2.3, 2.8] days per patient. This result has direct implications for hospital operations, as it implies improved resource utilization, fewer interruptions in elective care, and improved system responsiveness during peak demand periods.

Similarly to^59^ the average waiting time for patients in the high-priority group was reduced. In our case, from 43.5 [41.1, 45.7] to 31.3 [29.5, 33.0] days, an acceleration of approximately 12-days. This improvement reinforces the intended effect of our model: to ensure that patients with the highest multidimensional need receive an earlier surgical intervention, based on objective and expert-informed criteria.

In summary, the updated simulation results confirm that our hybrid AHP + K-Means methodology not only enhances equity and transparency in scheduling decisions but also generates tangible clinical and operational benefits. The incorporation of confidence intervals lends credibility and robustness to our findings, underscoring the value of the model as a scalable decision support tool for prioritization in high-demand hospital environments.

### Comparison with alternative prioritization models

Compared to the urgency-based prioritization approach previously developed by our team,^11^ the proposed model demonstrated greater specificity (AUC: 0.81 vs. 0.74) and achieved greater reductions in risk-adjusted surgical delay. This suggests that the integration of weighted criteria with unsupervised clustering offers a better alignment between predicted urgency and actual clinical deterioration.

## Discussion

Our proposed hybrid methodology, which combines AHP with K-Means clustering, offers a novel and clinically interpretable framework for surgical prioritization, which was designed and implemented within an ENT unit. One of the key strengths of this approach lies in its ability to integrate heterogeneous clinical and psychosocial variables into a single evidence-based prioritization score.

Through the use of expert-informed weighting schemes and normalization procedures, we were able to operationalize qualitative assessments and subjective experiences into quantitative values with consistent interpretation between patients. This ensures transparency and traceability in how decisions are made while maintaining fidelity to clinical judgment. The subsequent clustering phase allowed us to segment the population into internally coherent and clinically meaningful groups, demonstrating strong discriminatory power and alignment with expert expectations.

When compared to the standard first-in, first-out scheduling model, our simulations showed measurable improvements in critical dimensions: reduction in clinical risk, emergency hospitalization rate, and utilization of hospital beds. Importantly, we incorporated Monte Carlo simulations and 95 % Confidence Intervals to ensure the statistical robustness of these findings, thus increasing their generalizability in clinical practice. Although the magnitude of impact may vary depending on institutional constraints, our results indicate that even modest gains can be of considerable significance in high-complexity settings.

We included variables such as the capacity to study and the type of residence area in an exploratory capacity, with the aim of identifying possible structural inequities in access to care. These variables were not used independently to drive prioritization decisions, but rather to surface latent patterns of disadvantage that might otherwise remain invisible in purely clinical models. Their inclusion was reviewed and approved by the institutional Scientific Ethics Committee (Evaluation Report n° 09/2025), and interpreted in accordance with established principles of fairness and equity from the algorithmic bias literature.^39^ To further ensure ethical alignment, we conducted a fairness audit by analyzing demographic parity between priority groups. No evidence of systemic disadvantage was found (*p* > 0.10) for either socioeconomic status or rural residence. We believe that these variables can serve as diagnostic tools to inform an ongoing review of prioritization criteria ‒ not as determinants of care allocation.

However, we acknowledge certain limitations in our methodology. First, the AHP relies on subjective expert judgment, which, although structured and averaged between raters, may still be influenced by implicit biases. Second, while K-Means clustering provided strong performance in this case, its reliance on spherical distance metrics and fixed *k* selection may limit flexibility in populations with non-linear or overlapping characteristics. Additionally, although we modeled dynamic clinical risk using stochastic processes, the real-world progression of disease is subject to non-Gaussian fluctuations and unforeseen clinical events, which may not be fully captured in our simulations.

Compared to the existing literature, which often emphasizes single-variable prioritization rules or fixed categorical triage, our model advances the state-of-the-art by embedding multidimensional decision-making within a data-driven and scalable architecture. Other recent approaches have utilized supervised learning or optimization-based scheduling; however, these often require large volumes of labeled data and suffer from interpretability constraints. In contrast, our framework is well-suited to low-resource environments and enables straightforward calibration across different specialties or patient groups.

In summary, the AHP + K-Means methodology represents a clinically grounded, transparent, and adaptable strategy to prioritize patients on surgical waiting lists. Its combination of expert knowledge, unsupervised learning, and probabilistic simulation enables both operational efficiency and ethical responsiveness. Future research may extend this framework by incorporating real-time updates, adaptive feedback mechanisms, or reinforcement learning components to further enhance performance and responsiveness under uncertainty.

## Conclusions

In this study, we proposed and implemented a hybrid methodology that integrates AHP and K-Means clustering for surgical prioritization in the ENT unit of a high-complexity hospital in Chile. Although the hybrid AHP cluster has been established in prior literature, our contribution lies in its operationalization with ethical safeguards and real-world validation within a high-complexity ENT unit.

Our results provide strong empirical support for the effectiveness of this methodology. Through a series of cluster analysis and stochastic simulations, we demonstrated significant improvements in several key metrics: a 27 % reduction in mean clinical risk, a 41 % reduction in urgent hospitalizations, a 32 % reduction in urgent bed days, and an acceleration of more than 12-days in access for high-priority patients. These findings were further validated by confidence intervals derived from 1000 Monte Carlo iterations, underscoring the robustness and reliability of our estimates. The stratified distribution of scores and the consistency of the biopsychosocial profiles of the clusters reinforce the clinical validity of the model in the context of ENT care.

Despite these strengths, several areas warrant further refinement. The AHP step, while effective in incorporating expert knowledge, remains inherently subjective and may benefit from the inclusion of a broader and more diverse panel of stakeholders. Additionally, alternative clustering techniques such as DBSCAN or Gaussian Mixture Models could be explored to accommodate more complex, non-spherical distributions of patient profiles. Finally, our stochastic simulation model, although grounded in evidence and previous studies, assumes simplified distributions and may benefit from real-world calibration using longitudinal patient data from ENT surgical pathways.

The successful implementation of this model in practice will require alignment in the technical, clinical, and managerial domains. Hospital administrators must invest in systems capable of collecting and processing structured biopsychosocial data in real time. Equally critical is the participation of the ENT clinical team, whose participation and ongoing support are essential to maintain the relevance and accuracy of the model. This calls for a cultural shift toward prioritization schemes that are transparent, data-driven, and ethically grounded, particularly in resource-constrained healthcare systems.

To preserve transparency and ethical integrity over time, we will implement a biannual review of the selected variables and their associated weights. This process will be led by a multidisciplinary committee that includes clinicians, ethicists, and administrative stakeholders. All modifications will be version-controlled and documented to ensure institutional accountability and reproducibility.

Future research should focus on expanding the applicability of the model to other medical specialties and health conditions beyond otolaryngology, including the management of chronic diseases, transplantation, and coordination of outpatient care. Integrating real-time decision support tools, adaptive scheduling algorithms, or machine learning models capable of learning from feedback could further enhance the responsiveness and scalability of the model. We believe that our methodology offers a solid foundation for such advancements and represents a meaningful contribution to the field of patient-centered surgical planning and resource optimization, particularly within ENT services.

## Data availability statement

The data that support the findings of this study are available from the corresponding author upon request.

## Ethical considerations

In this research, we used anonymized secondary data from patients on the surgical waiting list, collected by attending physicians, and recorded in the hospital database. The methodology and the study were validated by the Scientific Ethics Committee of the Catholic University of Maule in Evaluation Report n° 09/2025. No direct interventions were performed, nor was personally identifiable information included. In fact, all patients gave their approval to the clinical team during routine medical care, so no additional written consent was required, according to local ethical standards and the Declaration of Helsinki. Since our research does not constitute a clinical trial, observational study, systematic review, meta-analysis, diagnostic or prognostic study, or animal research, the CONSORT, STROBE, PRISMA, STARD, and ARRIVE guidelines do not apply.

## Author's contribution

Conceptualization, F.S.-A.; Data curation, F.S.-A.; Formal analysis, F.S.-A., J.M., and V.D.; Funding acquisition, F.S.-A.; Investigation, F.S.-A., J.M., and V.D.; Methodology, F.S.-A., and J.M.; Project administration, F.S.-A.; Supervision, J.M., and V.D.; Writing-original draft, F.S.-A., and J.M.; Writing-review and editing, F.S.-A., J.M., and V.D. All authors have read and agreed to the published version of the manuscript.

## Funding

This research was funded by the “ANID Fondecyt Iniciacion a la Investigación 2024 n° 11240214”.

## Conflicts of interest

The authors declare no conflicts of interest.
